# A Pilot Study to Assess Suicidal Risk in Women Reporting Domestic Violence to a Law Enforcement Agency in South India

**DOI:** 10.1192/bjo.2024.259

**Published:** 2024-08-01

**Authors:** Lakshmi Keerthana Thatavarthi, Hari Priya Chintala

**Affiliations:** 1 St John's Medical College and Hospital, Bangalore, India; 2 Border Security Force, Siliguri, India

## Abstract

**Aims:**

In a recent national study in India, 35% of women reported experiencing domestic violence. The association between domestic violence and mental health outcomes especially suicidal risk has been less studied in Asia especially in India. With this context in mind, we aimed to establish a preliminary prevalence of suicidal risk in women reporting domestic violence using self-injurious thoughts and behaviors as proxy measures. We also wanted to probe the feasibility of assessing suicidal risk in a community center for vulnerable women with limited access to referral care and to determine the acceptability of safety plans as well as referral to a hospital setting for women with increased suicidal risk.

**Methods:**

A single center cross-sectional pilot study was conducted among 50 females who have officially reported Domestic Violence. The participants had reported this domestic violence to a ‘SHE Teams’ center in Telangana state, India, which is a women safety surveillance initiative launched by the state government. HARK (Humiliation, Afraid, Rape, Kick) questionnaire to assess the type of domestic violence experienced and SITBI (Self Injurious Thoughts and Behaviors Interview) questionnaire to evaluate the type of self-harm in victims were used.

**Results:**

It was found that 100% of the study population experienced emotional abuse, 50% sexual abuse, 74% physical abuse and 80% of them were afraid of their partners. It was also found that 64% had suicidal ideation, 40% had made a suicidal plan, 22% made suicidal gestures, 34% have attempted to commit suicide at least once. 12% had thoughts of Non-Suicidal Self Injury and 10% have committed Non-Suicidal Self Injury. Women who were unemployed and those who were harassed for dowry/endowment by the spouse or spouse's family had a statistically significant association with elevated suicidal risk. 17 participants were referred to a psychiatrist in the nearby hospital and 32 requested for shelter in fear of future violence.

**Conclusion:**

Domestic violence is a risk factor for poor mental health among women and suicide is one of the main causes of premature death in this population. To prevent more suicides in women, identifying risk and referral of domestic violence victims should be an essential part of health care systems apart from adequate legal support. This pilot study provides preliminary data for a future study of risk factors mediating suicidal risk in women who are victims of domestic violence and to develop targeted interventions as well.

